# Growth Temperature Alters the Thermal Sensitivity of Mesophyll Conductance in Wild and Cultivated Tomato

**DOI:** 10.1111/pce.70717

**Published:** 2026-07-02

**Authors:** Bar Ben Zeev, Adi Yaaran, Yotam Zait

**Affiliations:** ^1^ The Robert H. Smith Institute of Plant Sciences and Genetics in Agriculture, Faculty of Agriculture, Food and Environment The Hebrew University of Jerusalem Rehovot Israel

**Keywords:** activation energy, CO_2_ diffusion, mesophyll conductance, photorespiration, photosynthesis, *Q*_10_, *Solanum pennellii*, temperature response, thermal acclimation, tomato

## Abstract

Mesophyll conductance (*g*_m_) is a key component of internal CO_2_ diffusion and can limit photosynthesis in C_3_ leaves. Temperature responses of *g*_m_ are commonly treated as intrinsic properties of the leaf, yet it remains unclear whether they acclimate to growth conditions. Here, we tested whether growth temperature alters the thermal sensitivity of *g*_m_ and whether this differs between wild and cultivated tomato. We compared the wild species *Solanum pennellii* and the cultivated *Solanum lycopersicum* cv. M82 grown under four temperature regimes (16°C/10°C to 34°C/28°C, day/night). Using combined gas exchange and chlorophyll fluorescence, we quantified net photosynthesis (*A*_n_), *g*_m_ and photorespiration (*R*_*l*_). Growth temperature strongly altered the thermal sensitivity of *g*_m_ in both species. In M82, *g*_m_ *Q*_10_ increased from 0.92 to 1.29 across growth temperatures, and in *S. pennellii* from 1.03 to 1.44. Activation energy showed a similar shift, from values near zero under cold growth to 19.6 and 27.8 kJ mol^−1^ under warm growth. In contrast, species effects on *g*_m_ were small, whereas differences in *A*_n_ were larger and associated with variation in biochemical capacity. Thermal optima of *g*_m_ shifted with growth temperature in both species, while *R*_l_ remained consistently temperature sensitive with limited acclimation. The CO_2_ response of *g*_m_ were conserved across growth temperatures. These results show that the temperature sensitivity of *g*_m_ is not fixed, but acclimates to growth temperature, with a stronger effect than species identity. We conclude that *g*_m_ thermal sensitivity acclimates to growth temperature, but differences in photosynthetic performance are driven mainly by biochemical capacity rather than *g*_m_.

## Summary statement

Growth temperature acclimates the thermal sensitivity of mesophyll conductance in tomato leaves, increasing *g*_m_ temperature dependence in warm‐grown plants, while species differences in photosynthesis are driven mainly by biochemical capacity rather than *g*_m_.

## 1 Introduction

Photosynthesis is the primary process by which plants assimilate carbon. In C_3_ leaves, net CO_2_ assimilation (*A*_n_) depends on both biochemical reactions and the diffusion of CO_2_ from the atmosphere to the chloroplast (Farquhar and Sharkey 1982). Along this pathway, CO_2_ encounters several barriers, including stomatal conductance (*g*_sw_), and internal diffusion through the intercellular airspaces and mesophyll tissues, which together are described by mesophyll conductance (*g*_m_) and regulate CO_2_ transfer from the intercellular airspaces to the chloroplast stroma. Although the biochemical limitations of photosynthesis have been studied extensively, it is now clear that internal CO_2_ diffusion through mesophyll tissue can also impose a substantial constraint on carbon assimilation (Farquhar et al. 1980; Harley et al. 1992; Tomás et al. 2013; von Caemmerer and Evans 2015).

Mesophyll conductance is therefore a key component of the internal CO_2_ diffusion pathway. Early photosynthesis models assumed that *g*_m_ was sufficiently large to impose little limitation on photosynthesis (Farquhar et al. 1980), but this view has changed over the past two decades (Harley et al. 1992; Tholen et al. 2012; Evans 2021). A growing body of evidence shows that *g*_m_ strongly influences the CO_2_ concentration at the site of carboxylation. Mesophyll conductance reflects both structural and physiological properties of the leaf. Anatomical traits such as cell wall thickness, chloroplast positioning, and the surface area of mesophyll cells exposed to intercellular airspaces determine the internal diffusion pathway of CO_2_ (John et al. 1994; Terashima et al. 2011; Ellsworth et al. 2018). In addition, *g*_m_ is closely associated with photosynthetic capacity, photorespiration and stomatal conductance (Flexas et al. 2008). At the molecular level, proteins such as carbonic anhydrases and aquaporins have been proposed to facilitate CO_2_ transport within the mesophyll (Gillon and Yakir 2000; Li et al. 2020; Momayyezi et al. 2020). Despite this progress, the mechanisms regulating *g*_m_ under different environmental conditions remain poorly understood.

Temperature is a key environmental driver of photosynthesis (Sage and Kubien 2007). The response of net CO_2_ assimilation (*A*_n_) to temperature typically follows a parabolic pattern with a thermal optimum (*T*_opt_), above and below which assimilation declines (Berry and Bjorkman 1980). Many species can acclimate to growth temperature through shifts in *T*_opt_ or changes in the temperature sensitivity of underlying processes, allowing plants to maintain performance across different thermal environments (Way and Yamori 2014; Yamori et al. 2014).

Mesophyll conductance (*g*_m_) is also temperature dependent. Within a physiological range, *g*_m_ generally increases with leaf temperature, as observed in species such as tobacco and spinach (Bernacchi et al. 2002; Yamori et al. 2006), although both the magnitude and shape of this response vary widely among species (Scafaro et al. 2011; von Caemmerer and Evans 2015; Qiu et al. 2017). However, most studies have focused on short‐term, instantaneous responses to leaf temperature, whereas much less is known about how *g*_m_ acclimates to different growth temperatures. This distinction is important because thermal acclimation of photosynthesis is often interpreted in terms of biochemical changes, even though CO_2_ assimilation depends on CO_2_ diffusion from the atmosphere through stomata and internal diffusion within the leaf to the chloroplasts.

In particular, quantitative characterization of *g*_m_ thermal sensitivity, such as *Q*_10_ (the change in rate per 10°C increase in temperature) or activation energy (ΔHa, describing temperature dependence based on Arrhenius kinetics), as biochemical processes typically show stronger temperature dependence than physical CO_2_ diffusion processes (Bernacchi et al. 2002).

Understanding *g*_m_ acclimation is important because thermal acclimation of photosynthesis is typically interpreted through changes in *A*_n_, yet these responses integrate both biochemical capacity and CO_2_ diffusion. If *g*_m_ is relatively conserved, then shifts in *T*_opt_ of photosynthesis are likely driven primarily by biochemical processes. Conversely, if *g*_m_ acclimates strongly, it may play a more active role in shaping the temperature response of photosynthesis. Mesophyll conductance also exhibits characteristic short‐term responses to intercellular CO_2_ concentration (*C*_i_), typically remaining relatively stable near ambient conditions while declining at both low and high CO_2_ levels (Flexas et al. 2007; Márquez and Busch 2024; Tholen et al. 2012). Although this behaviour has been consistently reported across species, its underlying mechanism remains unresolved. Current evidence suggests that *g*_m_ responses to CO_2_ are only partially coordinated with stomatal conductance and may instead arise from internal processes within the leaf, including variable CO_2_ fluxes along the diffusion pathway and potential contributions from enzymatic facilitation (Mizokami et al. 2019; Márquez and Busch 2024). However, it remains unclear whether the CO_2_ dependence of *g*_m_ is modulated by environmental factors such as temperature, or whether it reflects conserved properties of the diffusion‐assimilation system. Addressing this question is critical for distinguishing between intrinsic regulation of *g*_m_ and emergent behaviour resulting from the coupling between CO_2_ diffusion and photosynthetic metabolism.

Wild relatives of crop species are widely used as sources of genetic variation for stress tolerance and physiological plasticity (Hajjar and Hodgkin 2007), although comparative studies show that domesticated cultivars can equal or even exceed their wild progenitors for some physiological traits in specific environments (Scafaro et al. 2011; Pelech et al. 2025). In tomato, wild species occupy diverse environments and display substantial variation in leaf structural traits that influence internal CO_2_ diffusion through the intercellular airspaces and mesophyll tissues (Muir et al. 2014). This raises the possibility that domestication has altered the regulation of *g*_m_ (Barbour et al. 2010; Scafaro et al. 2011; Tomeo and Rosenthal 2017). Selection for high photosynthetic capacity under optimal conditions may favour higher or less variable *g*_m_, whereas wild species may retain greater flexibility to cope with environmental variability. However, such flexibility may involve trade‐offs with structural traits associated with stress tolerance, such as increased leaf thickness or density, which can restrict CO_2_ diffusion (Niinemets et al. 2009; Tomás et al. 2013).

Here, we compared the temperature responses of CO_2_ assimilation and mesophyll conductance in two tomato species representing contrasting evolutionary backgrounds: the wild species *Solanum pennellii* and the cultivated tomato *Solanum lycopersicum* cv. M82 (Torgeman et al. 2024). Plants were grown under four temperature regimes (16°C/10°C, 22°C/16°C, 28°C/22°C and 34°C/28°C day/night) to test how growth temperature modifies the responses of *A*_n_ and *g*_m_. Using combined gas exchange and chlorophyll fluorescence measurements, we quantified CO_2_ assimilation, mesophyll conductance, stomatal conductance, photorespiration and biochemical parameters derived from CO_2_ response curves. This approach allowed us to assess the extent of *g*_m_ acclimation and determine whether domestication influences the thermal plasticity of internal CO_2_ diffusion as represented by *g*_m_.

## 2 Materials and Methods

### 2.1 Plant Material and Growth Conditions

The study was conducted in a controlled Phytotron greenhouse facility equipped with four independent day/night temperature‐controlled rooms set to 16°C/10°C, 22°C/16°C, 28°C/22°C and 34°C/28°C. Two tomato species were used: the cultivated *S. lycopersicum* cv. M82 and the wild species *S. pennellii*. Seeds were germinated at 28°C/22°C and seedlings were grown for 2 weeks under the same temperature regime in 4‐L pots filled with a growth medium composed of volcanic tuff, coconut coir and peat in varying proportions (Tuff Marom‐Golan, Ram 6, Israel). Plants were then transferred to the four temperature treatments and acclimated for 3 weeks prior to measurements. Plants were irrigated to full pot capacity twice daily to ensure complete soil saturation. Plants were grown under a long‐day photoperiod of 16 h light/8 h dark. Photosynthetic photon flux density (PPFD) at canopy height ranged between approximately 800 and 1200 µmol photons m^−2^ s^−1^ across the day. Light conditions were similar among the four temperatures‐controlled rooms. The irrigation solution contained Ca (2.0 ± 0.1 mM), Mg (1.2 ± 0.2 mM), NH_4_^+^ (0.27 ± 0.04 mM), NO_3_^−^ (4.6 ± 0.1 mM), P (0.30 ± 0.02 mM) and K (2.3 ± 0.2 mM), as previously described (Zait et al. 2017).

### 2.2 Gas Exchange and Chlorophyll Fluorescence Measurements

To quantify the temperature response of net photosynthesis (*A*_n_), stomatal conductance (*g*_sw_) and electron transport rate (*J*_t_) across growth temperatures, gas exchange and chlorophyll fluorescence were measured using a LI‐6800F portable photosynthesis system (LI‐COR Inc., Lincoln, NE, USA) equipped with a 6 cm^2^ fluorescence leaf chamber. Measurements were performed on the youngest fully expanded leaves that developed under the respective growth temperature. Unless otherwise stated, measurements were conducted at a PPFD of 1200 μmol photons m^−2^ s^−1^, a reference CO_2_ concentration of 420 μmol CO_2_ mol^−1^ air and a flow rate of 400 μmol air s^−1^. Relative humidity inside the chamber was maintained between 50% and 60%. Measurements were conducted under ambient oxygen concentration (21% O_2_), unless indicated otherwise. Additional measurements at 2% O_2_ were performed to support photorespiratory analyses and are presented in Supporting Material S1. Chlorophyll fluorescence was measured with the LI‐6800F chlorophyll fluorometer using the multiphase‐flash protocol, with a background actinic light of 1200 µmol photons m^−2^ s^−1^. The multiphase saturating flash consisted of three consecutive 300 ms phases (total duration 0.9 s) with a peak flash intensity of approximately 10 000 µmol photons m^−2^ s^−1^.

### 2.3 CO_2_ Response Curves

To characterize the response of *A*_n_ to intercellular CO_2_ concentration (*C*_i_) across growth temperatures, *A/C*_i_ curves were measured under light‐saturating conditions (PPFD 1200 μmol photons m^−2^ s^−1^). CO_2_ concentration was initially set to 420 μmol mol^−1^ and then decreased stepwise to 300, 200, 150, 100 and 50 μmol mol^−1^, followed by stepwise increases to 420, 600, 800, 1000, 1200 and 1500 μmol mol^−1^. Measurements at each step were recorded once gas exchange parameters stabilized. *V*_cmax_ and *J*_max_ were estimated in R using the fitaci function from the plantecophys package (Duursma 2015), with the bilinear fitting method and fitted *V*_*cmax*_ and *J*_*max*_ were normalized to 25°C for comparison across treatments. The *A*/*C*_i_ measurements were conducted inside the respective growth rooms without active control of leaf temperature, and leaf temperature (*T*_leaf_) recorded by the LI‑6800 at each CO_2_ step is provided for all treatments in Table S1.

### 2.4 Temperature Response Measurements

To characterize short‐term responses of leaf gas exchange to changes in leaf temperature, leaf temperature (*T*_leaf_) was controlled using the LI‐6800 temperature block. Measurements were initiated at 20°C and increased in 5°C increments up to 40°C. At each temperature step all parameters were recorded once steady conditions were achieved.

### 2.5 Estimation of Respiration in the Light, the Intercellular CO_2_ Compensation Point, Mesophyll Conductance and CO_2_ Photorespiratory Flux

To resolve internal CO_2_ diffusion and photorespiratory flux across leaf temperatures, respiration in the light (*R*_d_), the intercellular CO_2_ compensation point (*C*_i_^*^, the intercellular CO_2_ concentration at which net CO_2_ assimilation is zero in the light), mesophyll conductance (*g*_m_) and photorespiration (*R*_l_) were estimated from combined gas exchange and chlorophyll fluorescence measurements.

To quantify *R*_d_ and *C*_i_ *, Laisk curves were measured at three PPFD levels (400, 200 and 100 μmol photons m^−2^ s^−1^) (Figure S1). At each light level, CO_2_ concentration was decreased stepwise to 300, 200, 150, 100, 75, 50, 25 μmol CO_2_ mol air ^−1^. Because the fitted Laisk curves did not intersect at a single point, *C*_i_^*^ and *R*_d_ were estimated using a least‐squares optimization approach. Linear regressions were fitted to the linear portion of each Laisk curve, and the coordinates of the common intersection point were obtained by minimizing the sum of squared vertical distances between the candidate point and all fitted lines simultaneously.

*R*_d_ and *C*_i_^*^ were determined independently at each *T*_leaf_ across the full temperature range. Their temperature dependence was subsequently described using an exponential function (Table S1).

Mesophyll conductance to CO_2_ (*g*_m_) was calculated using the variable‐J method based on simultaneous gas exchange and chlorophyll fluorescence measurements (Harley et al. 1992; Loreto et al. 1992). *g*_m_ was calculated as:

$$g_{m}=\frac{A_{n}}{\left(C_{i}-C_{c}\right)}\;$$

where *C*_i_ is the intercellular CO_2_ concentration and *C*_c_ is the chloroplastic CO_2_ concentration. *C*_c_ was calculated as:

$$C_{c}=\frac{\left(\Gamma^{*}\cdot \;(J_{t}+8\;(A_{n}+R_{d}))\right)}{\left(J_{t}-4\;(A_{n}+R_{d})\right)}\;.$$

According to von Caemmerer et al. (1994), Γ* depends on *g*_m_ and *R*_d_, which are related to one another through the conductance between the intercellular airspace and the chloroplasts. However, because no independent *g*_m_ estimate was available a priori, we did not convert *C*_i_ * to Γ* and instead followed previous studies in treating *C*_i_ * from the Laisk analysis as a good approximation of Γ* (Harley et al. 1992; Walker and Ort 2015; Qiu et al. 2017).

*J*_t_ was derived from chlorophyll fluorescence following (Valentini et al. 1995) (Figure S2). Under non‐photorespiratory conditions (2% O_2_), a linear relationship between *ΦPSII* and *ΦCO*_2_ was established, and the resulting slope (*k*) and intercept (*b*) were used to calculate *J*_t_ at 21% O_2_ as:

$$J_{t}=\frac{\left(4\times (\Phi \mathrm{PSII}-b)\right)}{k}\times \mathrm{PPFD}$$

where PPFD is the incident photosynthetic photon flux density.

Photorespiratory CO_2_ release (*R*_l_), was calculated from *J*_t_, *A*_n_ and *R*_d_ according to Valentini et al. (1995):

$$R_{l}=\left(\frac{1}{12}\right)\;(J_{t}-4\;(A_{n}+R_{d})).$$

This formulation integrates gas exchange and fluorescence‐derived electron transport, allowing *R*_l_ to be resolved under each leaf temperature.

### 2.6 Light Response Curves

To assess the light dependence of *A*_n_ and *J*_t_ under photorespiratory and non‐photorespiratory conditions, light‐response curves were conducted in ambient (21% O_2_) and low oxygen (2% O_2_), while the CO_2_ concentration was fixed at 420 μmol CO_2_ mol^−1^ air. The measurement sequence involved light intensities of 2000, 1500, 1200, 1000, 800, 600, 400, 300, 200, 100 and 50 μmol photons m^−2^ s^−1^. Measurements at each light level were recorded once steady‐state gas exchange was achieved. These measurements were conducted inside the respective growth rooms without active control of leaf temperature, allowing leaf temperature to reflect the growth regime. These measurements were used to support the calibration of *J*_t_ and *R*_l_ (Figures S3, S4 and S5).

### 2.7 Thermal Response Modelling

To distinguish between long‐term acclimation to growth temperature and short‐term responses to changes in *T*_leaf_, thermal behaviour of *A*_n_, *g*_m_ and *R*_l_ was analysed using complementary empirical and thermodynamic approaches (Nobel 2009).

To determine the thermal optimum (*T*_opt_) of each parameter, second‐order polynomial models were fitted to the response of the parameter to *T*_leaf_, $\left(y=a\cdot {T_{\mathrm{leaf}}}^{2}\;+b{\cdot T}_{\mathrm{leaf}}\;+c\right)$. *T*_opt_ was calculated as −*b*/(2*a*) for each Species × growth temperature combination. The standard error of *T*_opt_ was calculated from the fitted model coefficients.

To quantify how the sensitivity of *A*_n_, *g*_m_ and *R*_l_ to temperature differed between species and growth regimes, activation energy (Δ*H*_a_) was calculated within the activation range (*T*_leaf_ ≤ 35°C). Only the temperature range showing a monotonic increase was included to avoid the influence of high‐temperature deactivation.

For each parameter, ln(parameter) was regressed against the inverse of absolute temperature (1/*T*) according to the Arrhenius relationship. In this linearized form, the slope of the regression equals −Δ*H*_a_/*R*. Activation energy (Δ*H*_a_) was therefore calculated directly from the slope as −slope × *R*, where *R* is the universal gas constant (8.314 J mol^−1^ K^−1^) and *T* is leaf temperature in Kelvin. Separate slopes were estimated for each Species × growth temperature combination using an interaction model. Thermal sensitivity of *g*_m_ was expressed as *Q*_10_. *Q*_10_ was estimated by fitting the model: $g_{m}=g_{m.25}\;\times \;Q_{10}^{\left(T_{\mathrm{leaf}}-25\right)/10}$directly to measured values between 20°C and 35°C using nonlinear least‐squares regression. The fitted *Q*_10_ parameter and its standard error were extracted from the model.

### 2.8 Statistical Analysis

All statistical analyses were performed in R version 4.3.2 (https://cran.r-project.org). Data are presented as means ± standard error (SE) unless otherwise stated. For gas exchange parameters (*A*_n_, *g*_m_ and *R*_l_), effects of Species and Growth Temperature were evaluated using linear models including their interaction. Species differences in parameters derived from *A*/*C*_i_ curves (*A*_max_, *V*_cmax_, *J*_max_ *;* Table 1) were tested using two‐sample *t*‐tests with Benjamini–Hochberg adjustment for multiple comparisons. For thermal activation analyses, full‐factorial linear models were fitted to ln(parameter) as a function of inverse temperature (1/*T*), Species, growth temperature, and their interactions, and Type III ANOVA was used to test the significance of Activation (Arrhenius slope), Species, Growth Temperature and their interactions. Statistical significance was defined at *p* < 0.05.

**Table 1:** Photosynthetic traits of *S. pennellii* and *S. lycopersicum* cv. M82 under four growth temperature regimes (16°C/10°C, 22°C/16°C, 28°C/22°C and 34°C/28°C day/night).

Trait	M82 (mean ± SE)	*Pennellii* (mean ± SE)	*p* (BH)
16°C/10°C			
*A*_max_ (µmol CO_2_ m^−2^ s^−1^)	25.9 ± 2.5	25.8 ± 1.2	0.970
*J*_max_ (µmol m^−2^ s^−1^)	157.1 ± 16.8	148.2 ± 5.3	0.640
*V*_cmax_ (µmol m^−2^ s^−1^)	99.9 ± 12.9	106.9 ± 3	0.630
22°C/16°C			
*A*_max_ (µmol CO_2_ m^−2^ s^−1^)	27.6 ± 3.2	33.1 ± 1.1	0.370
*J*_max_ (µmol m^−2^ s^−1^)	152.2 ± 18.2	165.9 ± 6.5	0.640
*V*_cmax_ (µmol m^−2^ s^−1^)	87.4 ± 10.6	97.9 ± 3.8	0.550
28°C/22°C			
*A*_max_ (µmol CO_2_ m^−2^ s^−1^)	33.3 ± 2	44.4 ± 1.8	0.041
*J*_max_ (µmol m^−2^ s^−1^)	152.9 ± 10.5	194.3 ± 15.3	0.290
*V*_cmax_ (µmol m^−2^ s^−1^)	75.9 ± 9	87.2 ± 5	0.550
34°C/28°C			
*A*_max_ (µmol CO_2_ m^−2^ s^−1^)	42.1 ± 0.9	43.9 ± 1	0.51
*J*_max_ (µmol m^−2^ s^−1^)	242.4 ± 13.5	246.4 ± 1	0.79
*V*_cmax_ (µmol m^−2^ s^−1^)	69.9 ± 6.8	72.6 ± 7.6	0.81

*Note:* Maximum CO_2_ assimilation rate (*A*_max_; µmol CO_2_ m^−2^ s^−1^), maximum electron transport rate (*J*_max_; µmol m^−2^ s^−1^) and maximum carboxylation capacity (*V*_cmax_; µmol CO_2_ m^−2^ s^−1^) were derived from *A/C*_i_ curves. Values are means ± standard error (*n* = 3–4). *p* values represent species differences from two‐sample *t*‐tests, adjusted for multiple comparisons using the Benjamini–Hochberg procedure.

## 3 Results

### 3.1 CO_2_ Response Curves and Derived Biochemical Parameters

Differences between species and growth temperatures were evident in the CO_2_ response curves (Figure 1) and the derived biochemical parameters (Table 1). *A*_max_ varied with growth temperature and was consistently higher in *S. pennellii*, with the largest interspecific differences observed at intermediate growth temperatures. For example, at 28°C/22°C, *A*_max_ was 33% higher in *S. pennellii* than in M82 (Table 1). *V*_cmax_ and *J*_max_ also varied with growth temperature, indicating adjustments in biochemical capacity (Table 1). This biochemical context is important for interpreting *g*_m_ responses, as it separates diffusion limitations from changes in carboxylation and electron transport capacity. Mesophyll conductance (*g*_m_) exhibited a non‐linear response to *C*_i_ in both species, increasing at low *C*_i_ and declining at higher *C*_i_ (Figure 1b), with peak values at 250–350 µmol mol^−1^. Differences between species were relatively small and decreased with increasing growth temperature, with *g*_m_ converging at high *C*_i_ across treatments. Photorespiration (*R*_l_) showed a distinct peak at low‐to‐intermediate *C*_i_ (~150–250 µmol mol^−1^) and declined at higher *C*_i_ (Figure 1c). Maximum *R*_l_ increased with growth temperature in both species, with the largest interspecific difference observed at 28°C/22°C.

**Figure 1:**
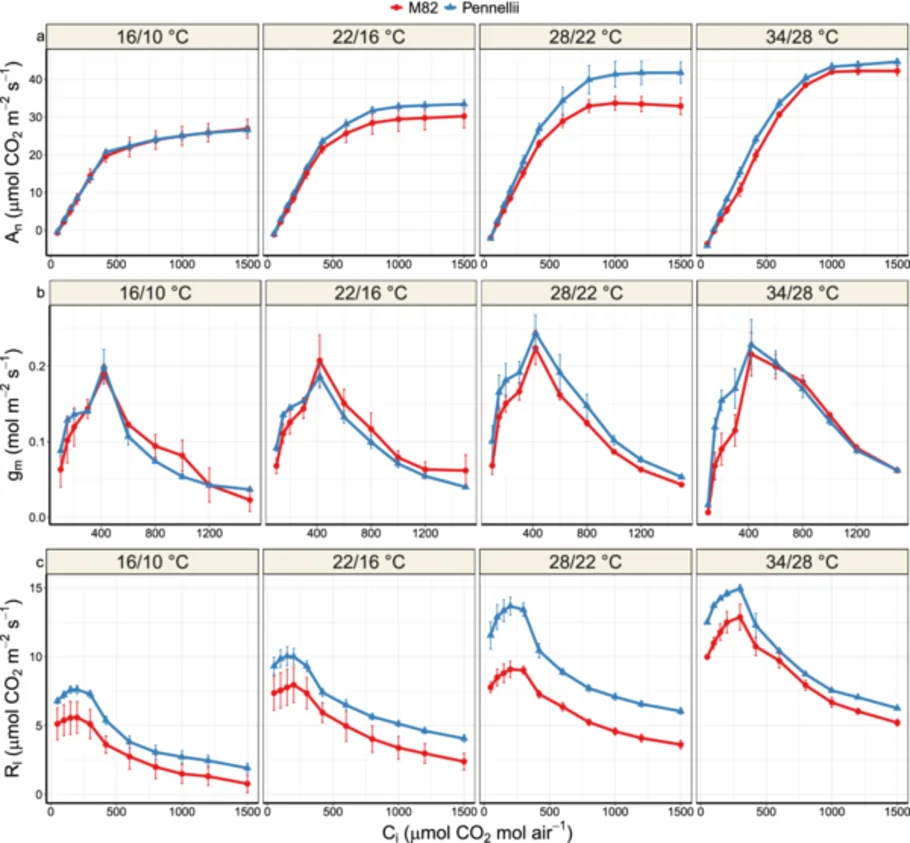
CO_2_ response curves of *S. pennellii* and *S. lycopersicum* (M82) under different growth temperatures. (a) Net assimilation of CO_2_ (*A*_n_; µmol CO_2_ m^−2^ s^−1^) in response to changes in the intercellular CO_2_ concentration (*C*_i_; µmol mol^−1^). (b) Mesophyll conductance to CO_2_ (*g*_m_; mol CO_2_ m^−2^ s^−1^) in response to *C*_i_. (c) Photorespiration rate (*R*_l_; µmol CO_2_ m^−2^ s^−1^) in response to *C*_i_. Measurements were conducted in two species of tomato: *S. pennellii* (blue) and *S. lycopersicum* (M82, red), grown under four growth temperature regimes of 16°C/10°C, 22°C/16°C, 28°C/22°C and 34°C/28°C (day/night). Values are means ± standard error (*n* = 3–4).

### 3.2 Response to Rapid Changes in Leaf Temperature (*T*_leaf_ Curves)

Net CO_2_ assimilation (*A*_n_), mesophyll conductance (*g*_m_), stomatal conductance (*g*_sw_) and photorespiration (*R*_l_) all varied with changes in leaf temperature (*T*_leaf_) across growth conditions (Figures 2 and 3). Measurements were conducted between 20°C and 40°C*. A*_n_ (Figure 3a) showed a parabolic response to increasing *T*_leaf_ in both species, increasing from 20°C to an intermediate optimum and declining at higher temperatures. The thermal optimum shifted with growth temperature, generally occurring around 25°C–30°C. Across treatments, *S. pennellii* maintained higher *A*_n_ than M82, particularly at warmer growth conditions. *g*_m_ (Figure 3b) increased with *T*_leaf_ from 20°C and reached a maximum at intermediate temperatures, followed by stabilization or a slight decline at higher temperatures. The position of the maximum varied with growth temperature and species, but overall patterns were similar between genotypes, with relatively small interspecific differences. *g*_sw_ (Figure 3c) showed a more variable response to *T*_leaf_ than *A*_n_ and *g*_m_, with no consistent pattern in the position of the maximum across growth temperatures. In general, the cultivated genotype (M82) exhibited a stronger increase in *g*_sw_ at higher temperatures, whereas *S. pennellii* maintained relatively high conductance across the temperature range. *R*_l_ (Figure 3d) increased strongly with increasing *T*_leaf_ in both species and reached its maximum at higher temperatures than *A*_n_ and *g*_m_. Across all growth conditions, peak *R*_l_ occurred at 35°C–40°C, indicating a stronger temperature dependence compared to photosynthesis and diffusion processes.

### 3.3 Thermal Optima (*T*_opt_) of Net CO_2_ Assimilation, Mesophyll Conductance and Photorespiration

Thermal optima (*T*_opt_) of *A*_n_, *g*_m_ and *R*_l_ were estimated from fitted temperature response models and increased with growth temperature in both species.

For *A*_n_, *T*_opt_ in M82 increased from 25.6°C at 16°C/10°C to 30.9°C at 28°C/22°C, followed by a slight decline to 29.8°C at 34°C/28°C. In *S. pennellii*, *T*_opt_ of *A*_n_ increased from 21.8°C to 29.6°C across the same range and remained lower than in M82 at 16°C/10°C (≈3.8°C) and 28°C/22°C (≈2.8°C).

*T*_opt_ of *g*_m_ showed a similar upward shift with growth temperature in both species. In M82, it increased from 27.0°C to 30.7°C, while in *S. pennellii* it increased from 27.2°C to 31.2°C, with only minor interspecific differences (≤ 0.7°C) across all growth conditions.

In contrast, *T*_opt_ of *R*_l_ was consistently higher than that of *A*_n_ and *g*_m_. In M82, *T*_opt_ of *R*_l_ increased from 34.1°C at 16°C/10°C to 39.2°C at 28°C/22°C and declined slightly to 37.6°C at 34°C/28°C. In *S. pennellii*, it increased from 34.3°C to 40.3°C across the same conditions, exceeding M82 at the highest growth temperature by ≈2.7°C.

### 3.4 Thermal Sensitivity of Net CO_2_ Assimilation, Mesophyll Conductance and Photorespiration (*Q*_10_)

Thermal sensitivity was assessed using *Q*_10_ calculated at 25°C. *Q*_10_ of *g*_m_ increased with growth temperature in both species. In M82, *Q*_10_ of *g*_m_ increased from 0.92 at 16°C/10°C to 1.29 at 34°C/28°C. In *S. pennellii*, *Q*_10_ of *g*_m_ increased from 1.03 to 1.44 across the same growth temperatures and was consistently higher than in M82 at 22°C/16°C, 28°C/22°C and 34°C/28°C (Figure 3 and 4). Under cold growth conditions, *g*_m_ showed low thermal activation (*Q*_10_ ≈ 1), whereas under warmer growth conditions *Q*_10_ increased markedly (Figures 4 and 5), indicating enhanced temperature sensitivity of *g*_m_. *Q*_10_ of *A*_n_ showed moderate variation across growth temperatures, ranging from 0.82 to 1.30 depending on species and treatment. In contrast, *Q*_10_ of *R*_l_ remained consistently high across all growth temperatures (1.84–2.32), indicating a stronger short‐term temperature sensitivity compared to *A*_n_ and *g*_m_.

### 3.5 Activation Energy and Arrhenius Model Effects

Activation energy (Δ*H*_a_; kJ mol^−1^) was estimated from Arrhenius models fitted separately for *A*_n_, *g*_m_ and *R*_l_ across species and growth temperatures. Positive Δ*H*_a_ values indicate thermal activation, whereas negative values indicate a decline in activity with increasing temperature.

Δ*H*_a_ varied strongly with both parameter and growth temperature. For *A*_n_, Δ*H*_a_ in M82 ranged from −6.5 at 16°C/10°C to 19.9 at 28°C/22°C, before decreasing to 8.66 at 34°C/28°C. In *S. pennellii*, Δ*H*_a_ values were lower overall, ranging from −15.0 at 16°C/10°C to 11.4 at 28°C/22°C and approaching zero (0.13) at 34°C/28°C. Positive and significant Δ*H*_a_ values for *A*_n_ were detected at 28°C/22°C in both species and at 34°C/28°C in M82.

In contrast, Δ*H*_a_ of *g*_m_ increased progressively with growth temperature in both species. In M82, Δ*H*_a_ shifted from −6.0 at 16°C/10°C to 19.6 at 34°C/28°C, while in *S. pennellii* it increased from 2.17 to 27.8 across the same growth temperatures. Δ*H*_a_ of *g*_m_ was not significantly different from zero under cold growth conditions but became positive and significant at 28°C/22°C and 34°C/28°C, particularly in *S. pennellii*.

Δ*H*_a_ of *R*_l_ was consistently high across all growth temperatures and showed little variation between species. In M82, Δ*H*_a_ ranged from 50.7 to 64.2 (declining to 48.3 at 34°C/28°C), while in *S. pennellii* it ranged from 49.0 to 62.5 (declining to 46.6 at 34°C/28°C). All Δ*H*_a_ estimates for *R*_l_ were highly significant, and interspecific differences were small relative to the magnitude of Δ*H*_a_.

Type III ANOVA of the Arrhenius models (Table 2) revealed parameter‐specific effects of Activation (Arrhenius slope), Species and Growth Temperature.

**Table 2:** Thermal response model effects based on Type III ANOVA of Arrhenius fits for net photosynthesis (*A*_n_; µmol CO_2_ m^−2^ s^−1^), mesophyll conductance (*g*_m_; mol CO_2_ m^−2^ s^−1^) and photorespiration rate (*R*_l_; µmol CO_2_ m^−2^ s^−1^).

	Activation energy (kJ mol^−1^)	Growth temperature	Species	Activation × Species	Species × Growth Temperature
*A*_n_ (µmol CO_2_ m^−2^ s^−1^)	0.084	< 0.001	< 0.001	0.001	< 0.001
*R*_l_ (µmol CO_2_ m^−2^ s^−1^)	< 0.001	< 0.001	< 0.001	0.065	0.036
*g*_m_ (mol CO_2_ m^−2^ s^−1^)	< 0.001	< 0.001	0.787	0.124	0.328

*Note:* Reported values represent *p* values for the effects of Activation (Arrhenius slope), Growth Temperature, Species, Activation × Species interaction and Species × Growth Temperature interaction.

For *A*_n_, significant effects of Species (*p* < 0.001) and Growth Temperature (*p* < 0.001) were detected, together with significant Activation × Species (*p* = 0.001) and Species × Growth Temperature (*p* < 0.001) interactions. These interactions indicate that the temperature sensitivity of *A*_n_ differed between species and varied with growth temperature.

For *g*_m_, significant effects of Activation (*p* < 0.001) and Growth Temperature (*p* < 0.001) were detected, whereas Species (*p* = 0.787) and Species × Growth Temperature (*p* = 0.328) were not significant. Activation × Growth Temperature was significant (*p* < 0.05), indicating that thermal activation of *g*_m_ depended primarily on growth temperature rather than species identity.

For *R*_l_, Activation, Species and Growth Temperature were all highly significant (*p* < 0.001). A Species × Growth Temperature interaction was also detected (*p* < 0.05), whereas Activation × Species was not significant. These results indicate strong temperature dependence of *R*_l_ with smaller but detectable interspecific differences.

## 4 Discussion

### 4.1 Growth Temperature Acclimates the Thermal Sensitivity of Mesophyll Conductance

This study shows that mesophyll conductance (*g*_m_) acclimates to growth temperature across a wide thermal range (Figures 1 and 2). Most previous work focused on instantaneous temperature responses of *g*_m_ under one or two growth conditions (Bernacchi et al. 2002; Yamori et al. 2006; Warren 2008; Scafaro et al. 2011; Walker et al. 2013; von Caemmerer and Evans 2015). Here, we show that *g*_m_ itself acclimates in its temperature sensitivity. Thermal optima (*T*_opt_) of *A*_n_, *g*_m_ and *R*_l_ increased with growth temperature in both species (Figure 3), consistent with the known shift in photosynthetic temperature optima with growth temperature (Berry and Bjorkman 1980; Yamori et al. 2014). Evidence for acclimation of *g*_m_ to growth temperature is limited and mostly based on studies with one or two growth conditions (Yamori et al. 2006), with little information on how *T*_opt_ of *g*_m_ changes across a wider growth temperature range. In our study, *T*_opt_ of *g*_m_ closely followed that of *A*_n_ and showed only minor differences between species (≤ 0.7°C), whereas differences in *A*_n_ were larger. In contrast, *T*_opt_ of *R*_l_ was consistently higher than that of *A*_n_ and *g*_m_ across all growth temperatures. These results indicate that shifts in *T*_opt_ represent a general acclimation response, but do not explain the differences in thermal sensitivity observed for *g*_m_, and therefore reflect a distinct component of temperature acclimation.

**Figure 2:**
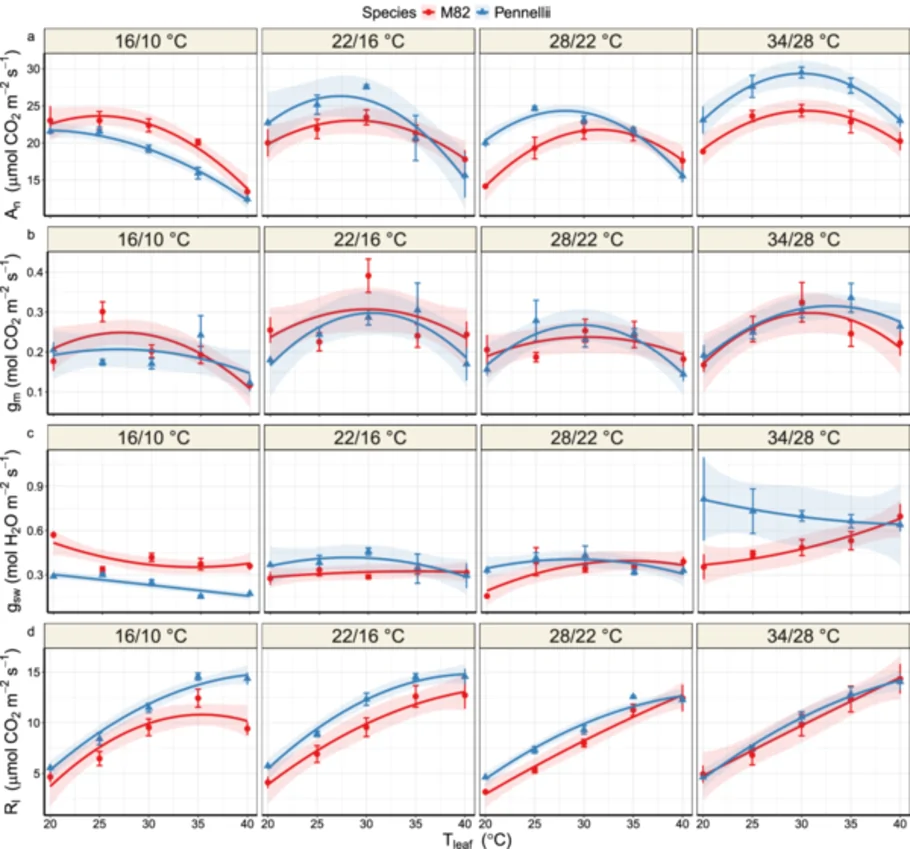
Response of net photosynthesis, mesophyll conductance, stomatal conductance and photorespiration to rapid changes in leaf temperature in *S. pennellii* and *S. lycopersicum* (M82). (a) Net photosynthesis (*A*_n_; µmol CO_2_ m^−2^ s^−1^), (b) mesophyll conductance (*g*_m_; mol CO_2_ m^−2^ s^−1^), (c) stomatal conductance (*g*_sw_; mol H_2_O m^−2^ s^−1^), and (d) photorespiration rate (*R*_l_; µmol CO_2_ m^−2^ s^−1^) in response to leaf temperature (*T*_leaf_). Measurements were conducted between 20°C and 40°C in two species of tomato: *S. pennellii* (blue) and *S. lycopersicum* (M82, red), grown under four growth temperature regimes of 16°C/10°C, 22°C/16°C, 28°C/22°C and 34°C/28°C (day/night). Solid lines represent fitted temperature response models and shaded areas represent model confidence intervals. Values are means ± standard error (*n* = 3). [Color figure can be viewed at wileyonlinelibrary.com]

**Figure 3:**
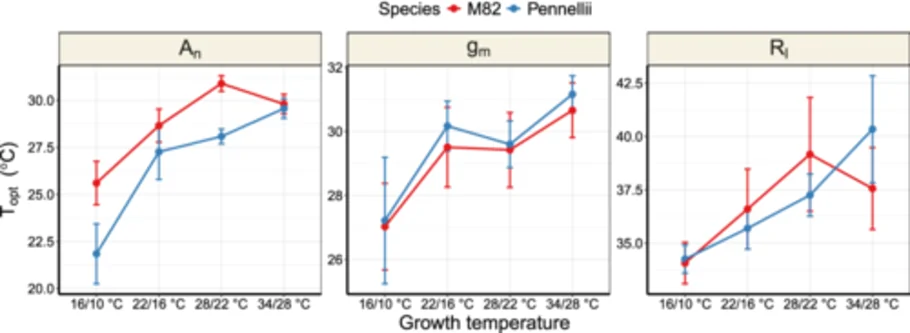
Thermal optimum (*T*_opt_) of net assimilation (*A*_n_), mesophyll conductance (*g*_m_) and photorespiration rate (*R*_l_) in *S. pennellii* and *S. lycopersicum* (M82). *T* (°C) values were calculated as $T_{\mathrm{opt}}=\;-b/\left(2a\right)$ from fitted quadratic temperature‐response models. Error bars represent ±SE estimated using propagation of uncertainty from the fitted coefficients a and b. Measurements were conducted in two tomato species, *S. pennellii* (blue) and *S. lycopersicum* (M82, red), grown under four growth temperature regimes of 16°C/10°C, 22°C/16°C, 28°C/22°C and 34°C/28°C (day/night). [Color figure can be viewed at wileyonlinelibrary.com]

The thermal sensitivity of *g*_m_ increased with growth temperature in both species (Figures 4 and 5). Under cold conditions, *g*_m_ showed weak temperature dependence (*Q*_10_ ≈ 1; Figure 4), whereas under warm conditions *Q*_10_ increased to 1.29 and 1.44 in M82 and *S. pennellii*, respectively. Similarly, Δ*H*_a_ increased from values close to zero or negative to 19.6 and 27.8 kJ mol^−1^ (Figure 5). These results show that acclimation affects the temperature response of *g*_m_, not only its magnitude (e.g., *Q*_10_ and Δ*H*_a_). Previous studies have reported positive responses of *g*_m_ to temperature, often following an Arrhenius relationship (Bernacchi et al. 2002), with some evidence for partial acclimation under contrasting growth conditions (Yamori et al. 2006). However, *g*_m_ temperature responses vary widely among genera and environmental conditions (von Caemmerer and Evans 2015; Qiu et al. 2017; Evans 2021). Differences between species have often been linked to structural traits, such as mesophyll cell wall thickness (Scafaro et al. 2011). and more recently to chloroplast surface area exposed to intercellular airspaces (Huang et al. 2022). While our study did not quantify anatomical traits or protein activity, the selective acclimation of *g*_m_ thermal sensitivity, with relatively small changes in its magnitude, and the similar responses observed in both species, suggests that these changes are driven by modifications in the properties of the diffusion pathway rather than by large structural differences (Shrestha et al. 2019). Candidate mechanisms include aquaporins, which can function as CO_2_ permeable membrane channels and influence CO_2_ diffusion (Uehlein et al. 2003; Hanba et al. 2004; Flexas et al. 2006), and carbonic anhydrase, which may modulate CO_2_/HCO_3_^−^ interconversion and thereby affect effective *g*_m_ (Gillon and Yakir 2000; Momayyezi et al. 2020).

**Figure 4:**
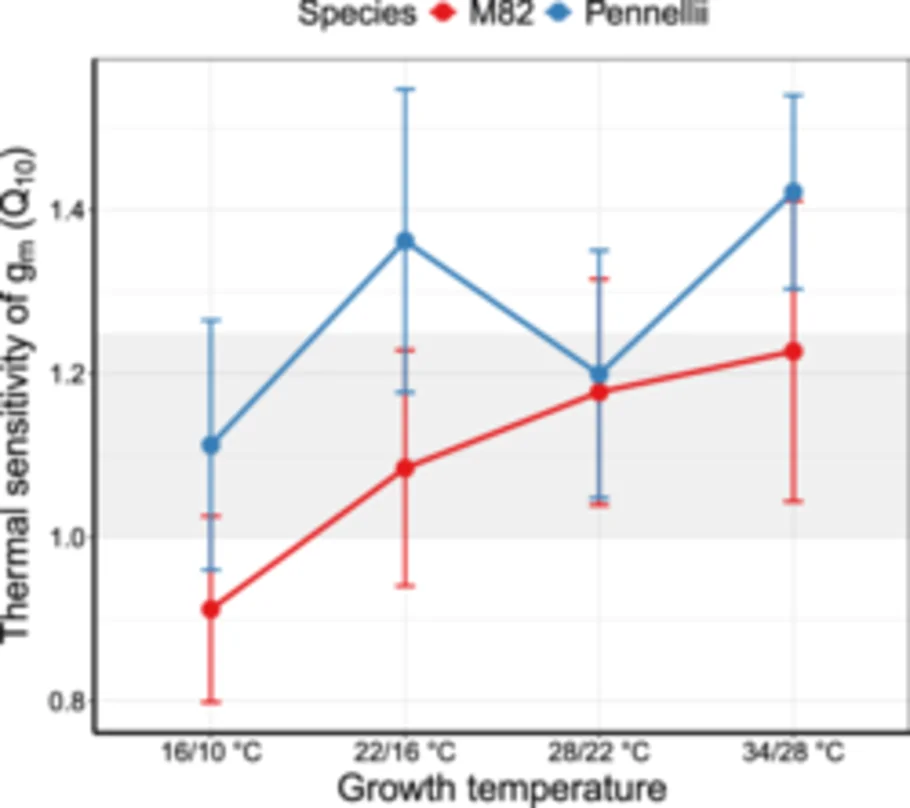
Temperature sensitivity expressed as *Q*_10_ of mesophyll conductance (*g*_m_) in *S. pennellii* and *S. lycopersicum* (M82). *Q*_10_ values were estimated by fitting the model $g_{m}=g_{m.25}\;\times \;Q_{10}^{\left(T_{\mathrm{leaf}}-25\right)/10}$ directly to measured *g*_m_ values between 20°C and 35°C. Points represent fitted *Q*_10_ estimates and error bars indicate ±SE of the fitted *Q*_10_ parameter. The grey shaded area indicates the range 1 < *Q*_10_ < 1.25, corresponding to low temperature sensitivity. Measurements were conducted in two tomato species, *S. pennellii* (blue) and *S. lycopersicum* (M82, red), grown under four growth temperature regimes of 16°C/10°C, 22°C/16°C, 28°C/22°C and 34°C/28°C (day/night). [Color figure can be viewed at wileyonlinelibrary.com]

**Figure 5:**
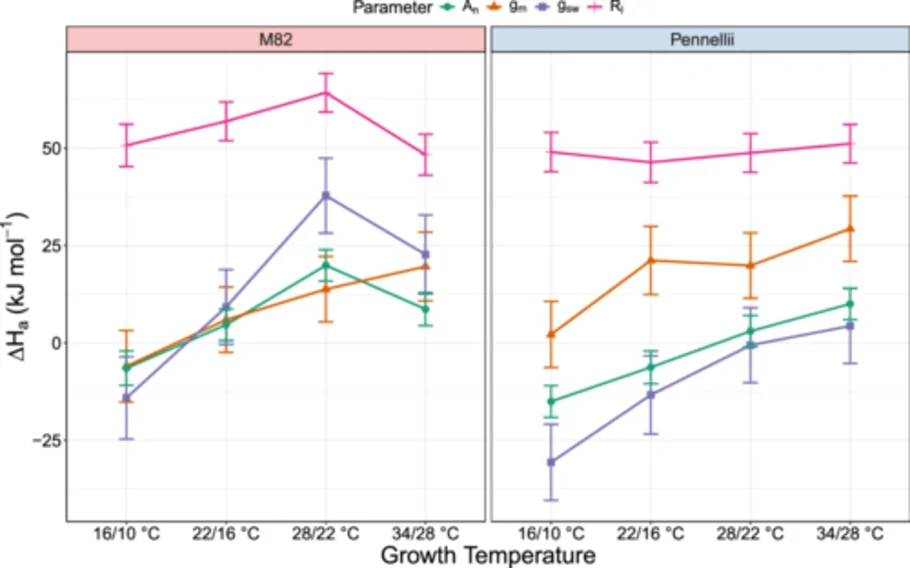
Activation energy (Δ*H*_a_; kJ mol^−1^) of net photosynthesis (*A*_n_), mesophyll conductance (*g*_m_), and photorespiration rate (*R*_l_) and stomatal conductance (*g*_sw_) estimated from Arrhenius fits across growth temperatures. Panels show species (M82 and *S. pennellii*), and line colours indicate the parameter (*A*_n_, orange; *g*_m_, green; *R*_l_, purple). Plants were grown under four growth temperature regimes of 16°C/10°C, 22°C/16°C, 28°C/22°C and 34°C/28°C (day/night). Values represent model‐derived estimates (mean ± SE). [Color figure can be viewed at wileyonlinelibrary.com]

Despite similarity in the acclimation of *g*_m_ thermal sensitivity in both species, the differences in net CO_2_ assimilation (*A*_n_) were much larger (Figure 2 and Table 2). *S. pennellii* maintained higher *A*_n_ under several growth temperatures (Figure 2a), while *g*_m_ remained relatively similar between species (Figure 2b). This indicates partial decoupling between diffusion capacity and photosynthetic performance as higher *A*_n_ in the wild species was supported primarily by biochemical capacity (*V*_cmax_ and *J*_max_; Table 1) rather than by large differences in *g*_m_. It is important to note that under cold growth (16°C/10°C) *S. pennellii* had lower *g*_sw_ than M82 (Figure 2c), so additional stomatal limitation of *A*_n_ in the wild species at low temperature cannot be excluded.

In soybean, Pelech et al. (2025) showed that a modern cultivated line exhibits approximately two‐fold higher mesophyll conductance than its wild ancestors, indicating that domestication and breeding have indirectly selected for increased *g*_m_ and reduced internal CO_2_ diffusion limitation, while also revealing substantial genotypic variation in *g*_m_ available to breeders. Together with studies showing genotype‐dependent trade‐offs between *g*_m_, photosynthetic capacity and water‐use efficiency in soybean (Tomeo and Rosenthal 2017), these results suggest that domestication can alter both the magnitude and plasticity of *g*_m_ in some crops, whereas in tomato our data indicate that wild‐cultivated differences in thermal performance arise mainly from biochemical plasticity despite relatively similar *g*_m_ across growth temperatures.

In contrast to *g*_m_, photorespiration (*R*_l_) remained strongly temperature sensitive across all growth conditions, with consistently high *Q*_10_ and Δ*H*_*a*_ (Figures 4, 5 and 5). This behaviour is consistent with the strong temperature dependence of Rubisco oxygenation and associated metabolic fluxes that contribute to photorespiratory CO_2_ release (Berry and Bjorkman 1980; Bernacchi et al. 2001). The contrasting patterns between *g*_m_ and *R*_l_ indicate that thermal acclimation differs between processes. *g*_m_ showed clear changes in temperature sensitivity, whereas *R*_l_ remained relatively stable. This highlights the need to separate diffusion from biochemical and respiratory/photorespiratory processes when interpreting thermal acclimation (Atkin and Tjoelker 2003; Way and Yamori 2014).

### 4.2 Decoupled Controls of Mesophyll Conductance Temperature and CO_2_ Responses

Mesophyll conductance exhibited a consistent peaked response to intercellular CO_2_ concentration across all growth temperatures (Figure 1b), remaining relatively stable at intermediate *C*_i_ and declining at both low and high CO_2_ levels. Notably, this pattern was conserved despite strong acclimation of gm thermal sensitivity, indicating that the processes governing the *g*_m_/*C*_i_ relationship are largely independent of those controlling its temperature response.

Previous work suggests that the CO_2_ dependence of gm reflects interactions between CO_2_ diffusion and internal carbon fluxes, including photorespiration and the balance between CO_2_ and HCO_3_^−^ along the diffusion pathway, rather than changes in the physical conductance itself (Flexas et al. 2007; Márquez and Busch 2024). The conservation of the *g*_m_/*C*_i_ response across temperatures in our study supports this interpretation and suggests that it reflects intrinsic properties of the diffusion‐assimilation system.

In contrast, the increase in *g*_m_ thermal sensitivity with growth temperature indicates that acclimation primarily affects the physical properties of the diffusion pathway, most likely membrane permeability and associated processes pathway (Bernacchi et al. 2002; Yamori et al. 2010). This suggests that CO_2_ and temperature responses of *g*_m_ are controlled by different processes. Given the rapid nature of *g*_m_ responses, such changes are unlikely to arise solely from structural modifications of the leaf, which occur over longer developmental timescales (Flexas and Diaz‐Espejo 2015). The underlying mechanisms remain unresolved.

## 5 Conclusions

Growth temperature modified the thermal sensitivity of mesophyll conductance across a wide thermal range. In both tomato species, *g*_m_ became more temperature sensitive in warm‐grown plants, demonstrating acclimation of internal CO_2_ diffusion through changes in *Q*_10_ and Δ*H*_a_. However, differences in photosynthetic performance between *S. pennellii* and M82 were much larger than differences in *g*_m_ and were supported mainly by variation in biochemical capacity (*V*_cmax_ and *J*_max_), indicating that *g*_m_ alone does not explain species differences in thermal performance. These results show that thermal acclimation of photosynthesis involves coordinated adjustments among diffusion, biochemistry and photorespiration. The acclimation observed here acts on the temperature sensitivity of *g*_m_ while leaving the *g*_m_/*C*_i_ response conserved, suggesting partially independent controls of CO_2_ and temperature‐dependent behaviour of internal diffusion.

## Supporting information

Supporting File

## Data Availability

The data that support the findings of this study are available from the corresponding author upon reasonable request.
