# Supplementary material for: Growth Temperature Alters the Thermal Sensitivity of Mesophyll Conductance in Wild and Cultivated Tomato
Source: Plant Cell Environ. 2026 Jul 2;49(11):7946–56. doi: 10.1111/pce.70717 (PMC13646658; doi:10.1111/pce.70717)
Supplement: Supplementary file 1 [file PCE-49-7946-s001.docx]

**Table S1. Leaf temperature (*T_leaf_*) of** Solanum pennellii **and** Solanum lycopersicum **cv. M82 grown under four temperature regimes (16/10, 22/16, 28/22, and 34/28°C day/night). Values are means ± standard error (SE).**

| **Growth Temperature** | **Species** | ***T_leaf_* (°C)** |
| --- | --- | --- |
| 16/10 °C | M82 | 22.40▒±▒0.10 |
|  | *S.Pennellii* | 23.24▒±▒0.11 |
| 22/16 °C | M82 | 26.45▒±▒0.19 |
|  | *S.Pennellii* | 26.84▒±▒0.15 |
| 28/22 °C | M82 | 31.88▒±▒0.26 |
|  | *S.Pennellii* | 32.37▒±▒0.15 |
| 34/28 °C | M82 | 36.86▒±▒0.31 |
|  | *S.Pennellii* | 38.77▒±▒0.26 |

**Figure S1.** CO_2_ response curves used for Laisk method analysis of net photosynthesis (*A_n_*) at multiple light intensities in *S. pennellii* and *S. lycopersicum* (M82) under different growth temperatures. Net CO_2_ assimilation rate (*A_n_*; µmol CO_2_ m⁻² s⁻¹) in response to intercellular CO_2_ concentration (*C_i_*; µmol CO_2_ mol⁻¹ air) measured at three light intensities (*Q*in▒=▒100, 200, and 500 µmol photons m⁻² s⁻¹; red, blue, and green, respectively). Panels represent four growth temperature regimes (16/10, 22/16, 28/22 and 34/28°C day/night) and two species (M82, upper panels; S. pennellii, lower panels).

**Figure S2** Relationship between *ΦPSII* and *ΦCO_2_* in *S. pennellii* and *S. lycopersicum* (M82). Effective quantum yield of PSII (*ΦPSII*) in response to changes in the quantum yield of CO_2_ assimilation (*ΦCO_2_*) measured under non-photorespiratory conditions (2% O_2_). Data are shown for two species of tomato: *S. pennellii* (blue) and *S. lycopersicum* (M82, red), together with a fit across all data (black). Linear fits were applied to the linear range of the data, and equations and R² values are shown for each species and for the combined dataset.

**Figure S3** Light response curves of net photosynthesis (*A_n_*) *in S. pennellii* and *S. lycopersicum* (M82) under different growth temperatures. Net CO_2_ assimilation rate (*A_n_*; µmol CO_2_ m⁻² s⁻¹) in response to incident light intensity (*Q_in_*; µmol photons m⁻² s⁻¹). Measurements were conducted in two species of tomato: *S. pennellii* (blue) and *S. lycopersicum* (M82, red), grown under four growth temperature regimes of 16/10, 22/16, 28/22 and 34/28°C (day/night). Values are means ± standard error (n▒=▒4).

**Figure S4** Light response curves of mesophyll conductance (*g_m_*) in *S. pennellii* and *S. lycopersicum* (M82) under different growth temperatures. Mesophyll conductance to CO_2_ (*g_m_*; mol CO_2_ m⁻² s⁻¹) in response to incident light intensity (*Q_in_*; µmol photons m⁻² s⁻¹). Measurements were conducted in two species of tomato: *S. pennellii* (blue) and *S. lycopersicum* (M82, red), grown under four growth temperature regimes of 16/10, 22/16, 28/22 and 34/28°C (day/night). Values are means ± standard error (n▒=▒4).

**Figure S5** Light response curves of stomatal conductance (*g_sw_*) in *S. pennellii* and *S. lycopersicum* (M82) under different growth temperatures. Stomatal conductance (*g_sw_*; mol H_2_O m⁻² s⁻¹) in response to incident light intensity (*Q_in_*; µmol photons m⁻² s⁻¹). Measurements were conducted in two species of tomato: *S. pennellii* (blue) and *S. lycopersicum* (M82, red), grown under four growth temperature regimes of 16/10, 22/16, 28/22 and 34/28°C (day/night). Values are means ± standard error (n▒=▒4).

**Figure S6** Light response curves of photorespiration rate (*R_l_*) in *S. pennellii* and *S. lycopersicum* (M82) under different growth temperatures. Photorespiration rate (*R_l_*; µmol CO_2_ m⁻² s⁻¹) in response to incident light intensity (*Q_in_*; µmol photons m⁻² s⁻¹). Measurements were conducted in two species of tomato: *S. pennellii* (blue) and *S. lycopersicum* (M82, red), grown under four growth temperature regimes of 16/10, 22/16, 28/22 and 34/28°C (day/night). Values are means ± standard error (n▒=▒4).

**Figure S7** Light response curves of net photosynthesis (*A_n_*) under non-photorespiratory conditions (2% O_2_) in *S. pennellii* and *S. lycopersicum* (M82) under different growth temperatures. Net CO_2_ assimilation rate (*A_n_*; µmol CO_2_ m⁻² s⁻¹) in response to incident light intensity (*Q_in_*; µmol photons m⁻² s⁻¹). Measurements were conducted in two species of tomato: *S. pennellii* (blue) and *S. lycopersicum* (M82, red), grown under four growth temperature regimes of 16/10, 22/16, 28/22 and 34/28°C (day/night). Values are means ± standard error (n▒=▒4).
